# Music reading experience modulates eye movement pattern in English reading but not in Chinese reading

**DOI:** 10.1038/s41598-022-12978-9

**Published:** 2022-06-01

**Authors:** Weiyan Liao, Sara Tze Kwan Li, Janet Hui-wen Hsiao

**Affiliations:** 1 Department of Psychology, University of Hong Kong, Hong Kong SAR, China; 2 Department of Social Sciences, School of Arts and Social Sciences, Hong Kong Metropolitan University, Hong Kong SAR, China; 3 The State Key Laboratory of Brain and Cognitive Sciences, University of Hong Kong, Hong Kong SAR, China; 4 The Institute of Data Science, University of Hong Kong, Hong Kong SAR, China

**Keywords:** Psychology, Human behaviour

## Abstract

Here we tested the hypothesis that in Chinese-English bilinguals, music reading experience may modulate eye movement planning in reading English but not Chinese sentences due to the similarity in perceptual demands on processing sequential symbol strings separated by spaces between music notation and English sentence reading. Chinese–English bilingual musicians and non-musicians read legal, semantically incorrect, and syntactically (and semantically) incorrect sentences in both English and Chinese. In English reading, musicians showed more dispersed eye movement patterns in reading syntactically incorrect sentences than legal sentences, whereas non-musicians did not. This effect was not observed in Chinese reading. Musicians also had shorter saccade lengths when viewing syntactically incorrect than correct musical notations and sentences in an unfamiliar alphabetic language (Tibetan), whereas non-musicians did not. Thus, musicians’ eye movement planning was disturbed by syntactic violations in both music and English reading but not in Chinese reading, and this effect was generalized to an unfamiliar alphabetic language. These results suggested that music reading experience may modulate perceptual processes in reading differentially in bilinguals’ two languages, depending on their processing similarities.

## Introduction

Recent research has shown that visual expertise in one domain may influence processing in other domains that involve similar processes. For example, as compared with novices, car experts had longer searching time for a target face with concurrent car distractors^1^ and had more difficulties in recognizing cars with face distractors^2^ due to a shared holistic processing mechanism. In Chinese character recognition, simplified Chinese readers could generalize left side bias and analytic character processing of simplified Chinese characters to the processing of traditional Chinese characters due to similarities in global character structure^3,4^. Similarly, recent research has reported that music reading experience can modulate perceptual processes in word reading. Interestingly, it is shown to modulate perceptual processes in English word reading due to similarities in the perceptual processes involved, but not in Chinese character reading. More specifically, Chinese-English bilingual musicians had better English word naming performance than non-musicians when words were presented in the left visual field (LVF)/right hemisphere (RH) and the center^5^, and a larger visual span for English letter identification in the right visual field (RVF) than non-musicians^6^. These effects were not observed in Chinese character naming or identification, suggesting that the modulation effect may depend on processing similarities across perceptual expertise domains. More specifically, both grapheme-phoneme mapping in reading English words and note-to-pitch mapping in reading musical segments involve mapping individual visual components to individual sounds from left to right^7^. Consequently, music reading expertise may have facilitated the letter-by-letter, serial visual processing of English words that characterizes RH English word recognition^8^, and perceptual learning of letters and notes that are typically recognized in the RVF/LH due to the left-to-right reading direction and required analytic processing^9^. In contrast, Chinese character recognition does not involve left-to-right grapheme-phoneme conversion and is more RH-lateralized or bilateral than English word processing^10–13^, and Chinese can be read in all directions^14^. Consequently, the facilitation effects from music reading expertise were not observed. These findings are consistent with the recent literature suggesting that transfer or modulation effects of perceptual expertise depend on the similarities in the perceptual representations and processes involved^3,4,9,15^.

While previous research has reported differential modulation effects of music reading expertise on visual word processing and visual span between English reading and Chinese reading in Chinese-English bilinguals, it remains unclear whether similar differential modulation effects can be observed in visual processing during sentence reading due to similarities and dissimilarities in perceptual demands among reading music notations, English sentences, and Chinese sentences. These modulation effects in visual processing may be reflected in eye movement planning behaviour during reading. More specifically, Chinese sentence reading differs from English sentence/music notation reading in its perceptual processing demands. Whereas both music notations and English sentences consist of musical segments/words separated by spaces, Chinese sentences do not have word boundaries. Also, musical segments and English words both consist of horizontally arranged symbols from left to right, each of which maps to a component in the pronunciation/sound. In contrast, components in a Chinese character can appear in different configurations and do not typically match components in the pronunciation. Thus, planning where to look during music reading may share higher similarities to English reading than Chinese reading.

In addition to perceptual processes, eye movement planning during sentence reading is related to the underlying language processes^16^. In particular, recent research has suggested that music training enhances sensitivity to regularities in sentence structure during language processing; this enhanced sensitivity may potentially influence eye movement planning behaviour during sentence reading. For example, Schon and colleagues showed that musicians outperformed non-musicians in detecting incongruities at the end of both musical phrases and French sentences^17^. Indeed, both language and music learning involve the understanding of sentences/music notations according to syntactic rules, which requires statistical learning of structural regularities through exposure^18^. The implicit knowledge of these regularities modulates how the stimuli are processed. For example, Waters and colleagues showed that musicians responded faster to rhythmically coherent musical segments than randomized ones, whereas non-musicians did not^19^. Similarly, in text reading, readers who had more experience with object relative clauses responded faster to sentences with object relatives than with subject relatives^20^. Recent research has suggested that music and language may share similar syntactic processing mechanisms. For example, in musicians, linguistic and musical incongruities elicited similar ERP P600 responses^21^. In typically developing children, music chord sequences with irregular endings elicited specific ERP components related to syntactic and harmonic integration that were not observed in children with specific language impairment^22^. These results suggested a strong association between the processing of linguistic and musical syntax.

Music expertise is also shown to modulate semantic processing in language. For example, a pervious study showed that musicians outperformed non-musicians in identifying animal-related words^23^. Another study found that musicians made fewer mistakes than non-musicians in judging whether a newly-learned word was semantically related to a presented picture^24^. Thus, music expertise may modulate sensitivity to both syntactic and semantic regularities, and in bilinguals, this effect may be observed in both of their two languages.

In Chinese-English bilinguals, both English and Chinese reading involve statistical learning of structural regularities similar to music reading. Thus, bilingual musicians’ enhanced sensitivity to structural regularities may be observed in both languages. More specifically, as compared with non-musicians, violations in these regularities may affect musicians’ language processes more, resulting in longer reading time in both languages. In contrast, since eye movement planning behaviour could be influenced by both linguistic and perceptual factors^16^, we speculated that this enhanced sensitivity to structural regularities of sentences may affect eye movement planning behaviour (i.e., where to look and the order of where to look) in English reading more than in Chinese reading due to the higher similarities in perceptual demands between English and music reading mentioned above. Thus, this effect in English reading may be reflected in overall eye movement planning pattern that includes fixation locations and the order of the fixation locations. In addition, previous research has suggested that bilingual musicians had increased visual span in English letter but not Chinese character identification^6^. Accordingly, the effect in English reading may also be reflected in saccade length.

To test this hypothesis, here we recorded Chinese-English bilingual musicians’ and non-musicians’ eye movements when reading English and Chinese sentences with different levels of linguistic regularity. We expected that in English reading, both participants’ reading fluency, eye movement pattern, and saccade length may be compromised by linguistic irregularity, and this effect might be larger in musicians than non-musicians due to musicians’ higher sensitivity to structural irregularities. In contrast, in Chinese reading, musicians and non-musicians may not differ in eye movement pattern or saccade length in response to structural irregularities due to dissimilarities in perceptual demands between music notation and Chinese reading. In addition, we included both music notation and Tibetan sentence stimuli to examine participants’ performance and behaviour in reading stimuli where only musicians had experience with (music notations), and an unfamiliar alphabetic language where neither participant group had experience with (Tibetan sentences). We hypothesized that in reading music notations, musicians and non-musicians would differ in reading fluency and eye movement planning behaviour due to their difference in music reading experience. In contrast, they would not differ in viewing Tibetan sentences.

## Methods

### Participants

Participants were 86 Chinese (L1)-English (L2) bilinguals grew up and received standard education in Hong Kong. Their age ranged from 18 to 34 (*M* = 21.45, *SD* = 2.98). They had similar college education backgrounds. They were categorized as musicians (*n* = 43; 21 males), who were well-trained pianists and proficient in reading music notations, and non-musicians (*n* = 43; 21 males), who did not receive any formal music training and reported unable to read music notations. The two groups did not differ significantly in age, *t*(84) = 1.343, *p* = 0.183. A power analysis showed that a sample size of 86 was needed to acquire a small to medium effect size (η_p_^2^ = 0.03) in a within-between interaction test using ANOVA with 95% power and 0.05 alpha.

Musicians and non-musicians were matched in handedness (Edinburgh Handedness Inventory^25^), *t*(84) = −0.809, *p* = 0.421; language exposure as self-reported English reading hours per week, *t*(84) = 0.840, *p* = 0.403, and Chinese reading hours per week, *t*(84) = -0.824, *p* = 0.412; English proficiency (LexTALE^26^), *t*(84) = 1.360, *p* = 0.178; Chinese proficiency by grades in the matriculation public examination of Chinese Language (HKCEE/HKALE/HKDSE; scores were converted into a 7-point scale), *t*(83) = −1.063, *p* = 0.291; and familiarity with Tibetan letters (a 10-point Likert scale), *t*(84) = 0.721, *p* = 0.473. In verbal and visuospatial working memory, they did not differ in reaction time (RT) of a verbal two-back task, *t*(84) = -0.625, *p* = 0.533, or accuracy, *t*(84) = 0.939, *p* = 0.351, and RT, *t*(84) = -0.075, *p* = 0.940, of a visuospatial two-back task^27^. Musicians had higher accuracy in the verbal two-back task, *t*(84) = 2.223, *p* = 0.029, *d* = 0.479. This measure thus was added as the covariate in the analyses reported here. All participants started learning English as a second language at age 3 at kindergarten (the standard curriculum in Hong Kong). No participants had experience with Tibetan.

We also used the self-reported inventory Goldsmiths Musical Sophistication Index (Gold-MSI^28^) to examine participants’ musical sophistication (Gold-MSI subscales had fairly high reliability^28^). Musicians had higher MSI than non-musicians in all MSI indices: active engagement, *t*(84) = 9.82, *p* < 0.001, *d* = 2.118; perceptual abilities, *t*(75.3) = 8.38, *p* < 0.001, *d* = 1.808; musical training, *t*(84) = 13.46, *p* < 0.001, *d* = 2.902; emotions, *t*(84) = 7.14, *p* < 0.001, *d* = 1.540; singing abilities, *t*(84) = 8.25, *p* < 0.001, *d* = 1.780. Table 1 summarises descriptive statistics of the participants.

**Table 1:** Descriptive statistics of the participants.

	Music		Non-musician		Total	
	Mean	SD	Mean	SD	Mean	SD
Age (years)	21.88	3.50	21.02	2.32	21.45	2.98
Handedness (-100 to 100)	57.44	35.18	63.49	34.10	60.47	34.57
English reading hour/week	16.87	13.66	14.54	12.01	15.70	12.84
Chinese reading hour/week	16.78	16.19	19.77	17.48	18.28	16.82
English proficiency (0 to 1)	0.74	0.11	0.71	0.11	0.73	0.11
Chinese proficiency (1 to 7)	4.67	1.68	5.02	1.41	4.85	1.55
Familiarity with Tibetan letters (1 to 10)	1.26	0.69	1.16	0.48	1.21	0.60
Verbal two-back accuracy	0.83	0.17	0.74	0.20	0.79	0.19
Verbal two-back RT (ms)	920.20	183.65	944.74	180.22	932.47	181.29
Visuospatial two-back accuracy	0.76	0.17	0.72	0.18	0.74	0.18
Visuospatial two-back RT (ms)	928.41	206.68	931.35	149.85	929.88	179.45
MSI active engagement (9–63)	34.93	6.11	20.72	7.26	27.83	9.78
MSI perceptual abilities (9–63)	49.67	7.09	33.88	10.11	41.78	11.77
MSI musical training (7–49)	14.45	3.30	5.58	2.79	10.02	5.40
MSI emotions (7–49)	32.14	5.00	24.77	4.56	28.45	6.03
MSI singing abilities (6–42)	33.09	6.19	20.37	7.99	26.73	9.56

The experiment was approved by the Human Research Ethics Committee of the University of Hong Kong (HREC reference number: EA1702010). All experiments were performed in accordance with the American Psychological Association ethical standards. All participants gave their informed consent prior to their inclusion in the study.

### Materials

The materials consisted of English sentences, Chinese sentences, musical phrases as expertise stimuli for musicians, and Tibetan sentences as control stimuli which no participants had reading experience with. English and Chinese stimuli consisted of three sentence types differing in structural regularity: original, semantically incorrect, and random word list. Musical and Tibetan stimuli consisted of two conditions: original and random segment/syllable list, since musical phrases do not carry specific semantic meanings^7^, and no participant read Tibetan stimuli. Each condition had 24 stimuli.

**Figure 1:**
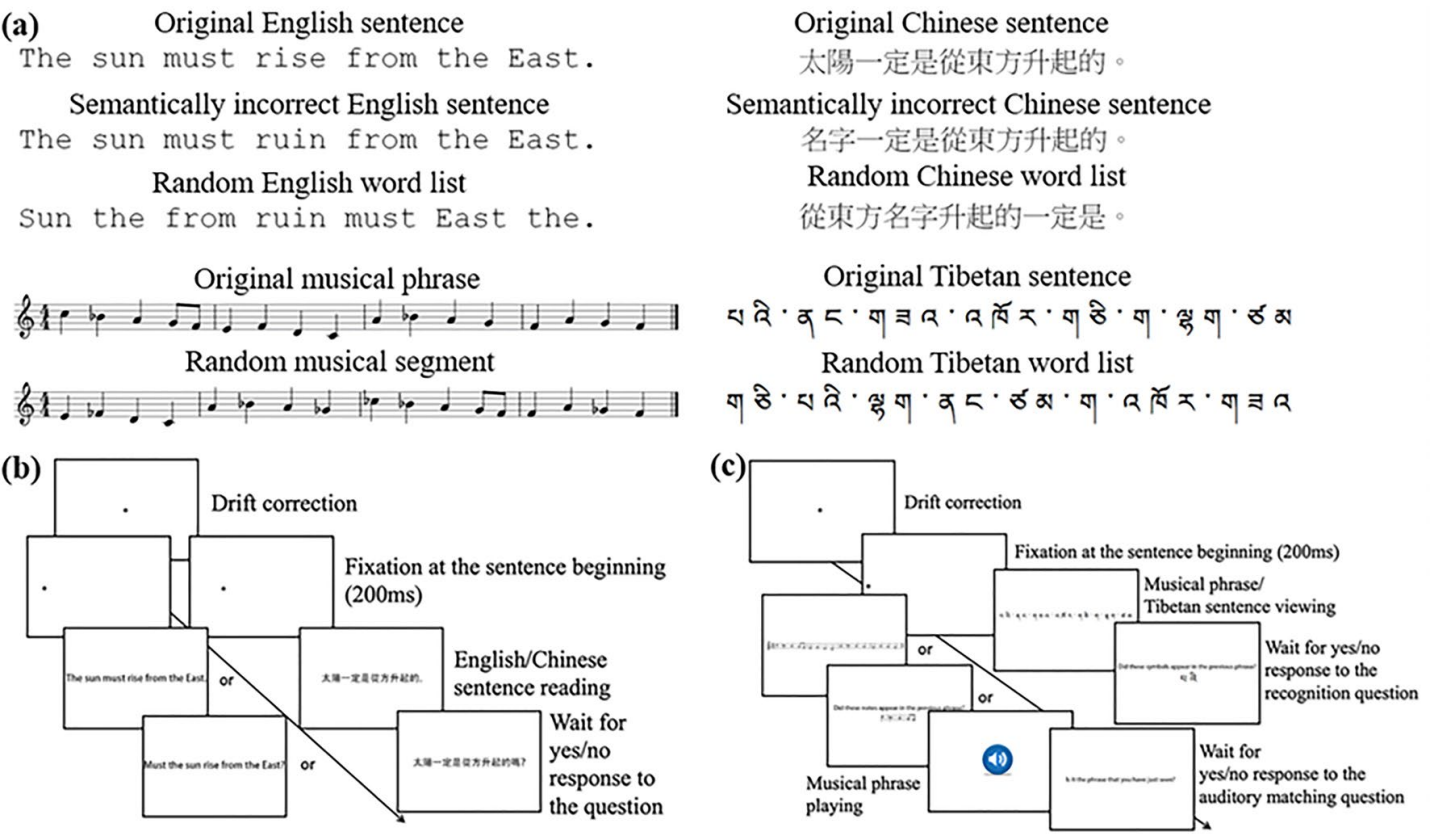
(**a**) Upper left are sample English stimuli; upper right are sample Chinese stimuli; lower left are sample musical stimuli; lower right are sample Tibetan stimuli. (**b**) Procedure of the English/Chinese reading task. (**c**) Procedure of the musical phrase/ Tibetan sentence viewing task.

In English, original sentences with a neutral valence were selected from an English learning website^29^. Each sentence consisted of 5 to 7 words, with 2 to 11 letters in each word. Semantically incorrect sentences were created by replacing a target word from each of the original sentences with another word of the same grammatical type, word length, and similar word frequency (SUBTLEX^30^). The target word was selected based on its position in a sentence: we selected an equal number of target words from the beginning, middle, or ending sections of the sentences. Random word lists were created by randomly rearranging the word order of the semantically incorrect sentences such that the words did not follow any syntactic rules (Fig. 1a). All sentences were in the same visual length. Under a viewing distance of 61 cm, each letter subtended a horizontal and vertical visual angle of 0.384° × 0.384°; each English sentence subtended 12.48° × 0.56°.

In Chinese, original sentences were translated from English stimuli and validated by three native Chinese readers. Each sentence consisted of 1-to-3-character words and was 11 characters in length. Each character had nine strokes on average. Semantically incorrect sentences and random word lists were created in the same way as English stimuli (Fig. 1a). Word/character frequency information were obtained from Chinese databases^31,32^. Each Chinese character subtended a horizontal and vertical visual angle of 0.573° × 0.573°; each Chinese sentence subtended 7.05° × 0.56°.

In musical stimuli, four-bar phrases were selected from 21 four-part chorales (SATB vocal repertoires) by J. S. Bach. All phrases were in treble clef, 4/4 time, with diatonic keys (F, Bb, D, and G major) indicated through corresponding accidentals and ended in I or V chords, counterbalanced across phrases. The notes used ranged from two lower ledger lines (A3) to the fifth line (F#5). Random segments were created by randomly rearranging the bars from the original phrases. Unnecessary accidentals (sharps to phrases in G or D major/flats to F or Bb major respectively) were added randomly to create a non-diatonic, chromatic musical phrase that violated traditional diatonic chord progressions. All phrases were in the same visual length (Fig. 1a). Each music note subtended a horizontal and vertical visual angle of 0.384° × 1.907°. Each musical sentence, excluding the treble clef and time signature, subtended 18.58° × 1.41°.

In Tibetan, original sentences were selected from a Tibetan news website^33^. In Tibetan sentences, syllables are written from left to right, separated by tsek marks (i.e., the dots shown in Fig. 1a). A word may consist of one or multiple syllables, and most Tibetan words are monosyllabic. In our stimuli, each sentence consisted of 7 to 9 syllables, with 1 to 6 letters in each syllable. Random syllable lists were created by randomly rearranging the syllable order from the original sentences. The size of a Tibetan letter was defined using the letter 'ཟ', which subtended about a horizontal and vertical visual angle of 0.384° × 0.384°. Each Tibetan sentence subtended 18.58° × 1.41°. All sentences were in the same visual length as the musical phrase stimuli.

LexTALE was used to examine English proficiency. It has a moderate to good internal reliability (split-half reliabilities of average percentage of correct responses, Spearman-Brown corrected, was 0.814 in Dutch participants and 0.684 in Korean participants^26^).

Verbal and spatial two-back tasks were used to measure participants’ verbal and spatial working memory^27^.

### Design

For English and Chinese reading, the design consisted of one within-subject variable, sentence type (original vs. semantically incorrect vs. random word), and a between-subject variable, music expertise (musicians vs. non-musicians). The dependent variables were reading time, saccade length, and eye movement pattern as measured using Eye Movement analysis with Hidden Markov Models (EMHMM^34^). In addition to these eye movement measures focusing on eye movement pattern for testing our hypotheses, we included other common eye movement measures in reading research including fixation duration, regression rate (i.e., frequency of regressive saccades to a previous word during reading) and skipping rate (i.e., percentage of words skipped during reading) as an exploratory examination. Three planned comparisons were conducted: original vs. semantically incorrect, to examine semantic processing effect; semantically incorrect vs. random word, to examine syntactic processing effect; original vs. random word, to examine linguistic regularity effect (a combination of semantic and syntactic regularity). A similar design was used for musical phrase and Tibetan sentence viewing, except that sentence type had only two levels, original vs. random segment/syllable. ANCOVA with verbal two-back accuracy as a covariate was used. For each stimulus type, trials in different sentence type conditions were presented in one block with the trial order randomized, so that participants could not anticipate the condition.

The average luminance of stimuli was 3.44 cd/m^2^. With 82.5 cd/m^2^ background luminance, the Weber contrast of the stimuli was -0.96. All stimuli were presented in black with a white background on a 17’ CRT monitor with a resolution of 1280 × 960. Eye movements were recorded with an EyeLink 1000 eye tracker (SR Research Ltd.). Monocular tracking of the dominant eye in the pupil and corneal reflection tracking mode was used. A chinrest was used to reduce head movement. Calibration and validation were performed before each block; recalibration took place whenever drift correction error was larger than 0.5° of visual angle. EyeLink default settings for cognitive research were used in data acquisition (saccade motion threshold: 0.1°; saccade acceleration threshold: 8000°/s^2^; saccade velocity threshold: 30°).

### Procedure

Participants first completed a demographic and music background questionnaire, LexTALE, MSI, Edinburgh Handedness Inventory, and verbal and spatial two-back tasks. Then, participants completed English sentence reading, Chinese sentence reading, musical phrase viewing, and Tibetan sentence viewing tasks in separate blocks, with the block order counterbalanced across participants. Each trial started with a solid circle at the screen center for drift correction; recalibration was performed when the gaze position error was larger than 0.5° of visual angle. Afterwards, a dot was presented on the left side of the screen, and the participant was instructed to look at the dot. Once a 200-ms fixation was detected, the stimulus was presented at the center (Fig. 1b,c). In Chinese and English reading, participants read the sentence and answer a related question afterwards. In musical phrase and Tibetan sentence viewing, participants viewed the stimuli and perform a stimulus recognition task afterwards. They pressed the space bar when they finished reading/viewing the stimuli.

To examine reading efficacy, for English and Chinese sentences, they answered a comprehension question after reading an original sentence, or a word recognition question after reading a semantically incorrect sentence/random word list. In the word recognition task, the target word of a ‘no’ trial was selected from the corresponding original sentence. For Tibetan sentences and musical phrases, they answered a syllable/musical segment recognition question after viewing each stimulus, and the target syllable/segment was from the corresponding sentences/phrases across the two sentence type conditions. The same numbers of ‘yes’ and ‘no’ trials were included. For the musical phrase task, an auditory musical phrase matching task was carried out after the musical segment recognition task. Participants listened to an auditory musical phrase and judged whether it was identical to the visual stimulus they saw in the trial (Fig. 1c). This task served as an expertise task to examine whether musicians indeed had better abilities to match music notations to corresponding auditory musical phrases than novices and whether this expertise measure was associated with other expertise effects observed.

### Eye Movement analysis with Hidden Markov Models (EMHMM)

EMHMM^34^ was used to quantify a participant’s eye movement pattern, taking both temporal and spatial dimensions of eye movements into account. Using this approach, we summarized an individual’s eye movement pattern in a sentence type condition using a hidden Markov model (HMM, a type of time-series statistical model in machine learning), including person-specific regions of interest (ROIs) and transition probabilities among these ROIs. Parameters of an HMM were estimated from eye movement data. Thus, each participant had three HMMs, each corresponding to a sentence type. Then, we clustered all individual HMMs into two groups^35,36^ to reveal two representative eye movement patterns. The similarity between an individual’s eye movement pattern in a sentence type condition and a representative pattern could be assessed using the log-likelihood of the individual’s eye movement data being generated by the representative pattern HMM^37^. This quantitative measure of eye movement pattern allowed us to examine changes in eye movement pattern across sentence types and their association with music expertise.

When training individual HMMs, we set the range of possible number of ROIs to be 1 to 3 to capture participants’ general eye movement patterns that may involve looking at the beginning, middle, or end of the sentences/musical phrases. The use of 3 ROIs corresponded to about 2 words per ROI for English sentences, 3 to 4 characters (about 2 words) per ROI for Chinese sentences, 1 to 1.5 bars per ROI for musical phrases, and 2 to 3 words per ROI for Tibetan sentences. EMHMM uses a variational Bayesian approach to determine the optimal number of ROIs from the preset range for each model. Since sentences differed in number of words and word length, which could influence eye fixation locations during reading^38^, the use of maximum 3 ROIs could help discover a general eye movement pattern across all sentences and avoid capturing ROIs that were specific to a sentence. Each individual model with a different preset number of ROIs was trained for 100 times, and the model with the highest data log-likelihood was used. Following previous studies using EMHMM^39–51^, we clustered individual HMMs into two clusters to discover two representative patterns, so that each individual’s eye movement pattern could be quantified (using data log-likelihoods) along the dimension contrasting the two representative patterns. The number of ROIs for creating representative HMMs of the clusters was set to the median number of ROIs in the individual models. The clustering algorithm was run for 100 times; the result with the highest data log-likelihood was used.

## Results

### English sentences

**Figure 2:**
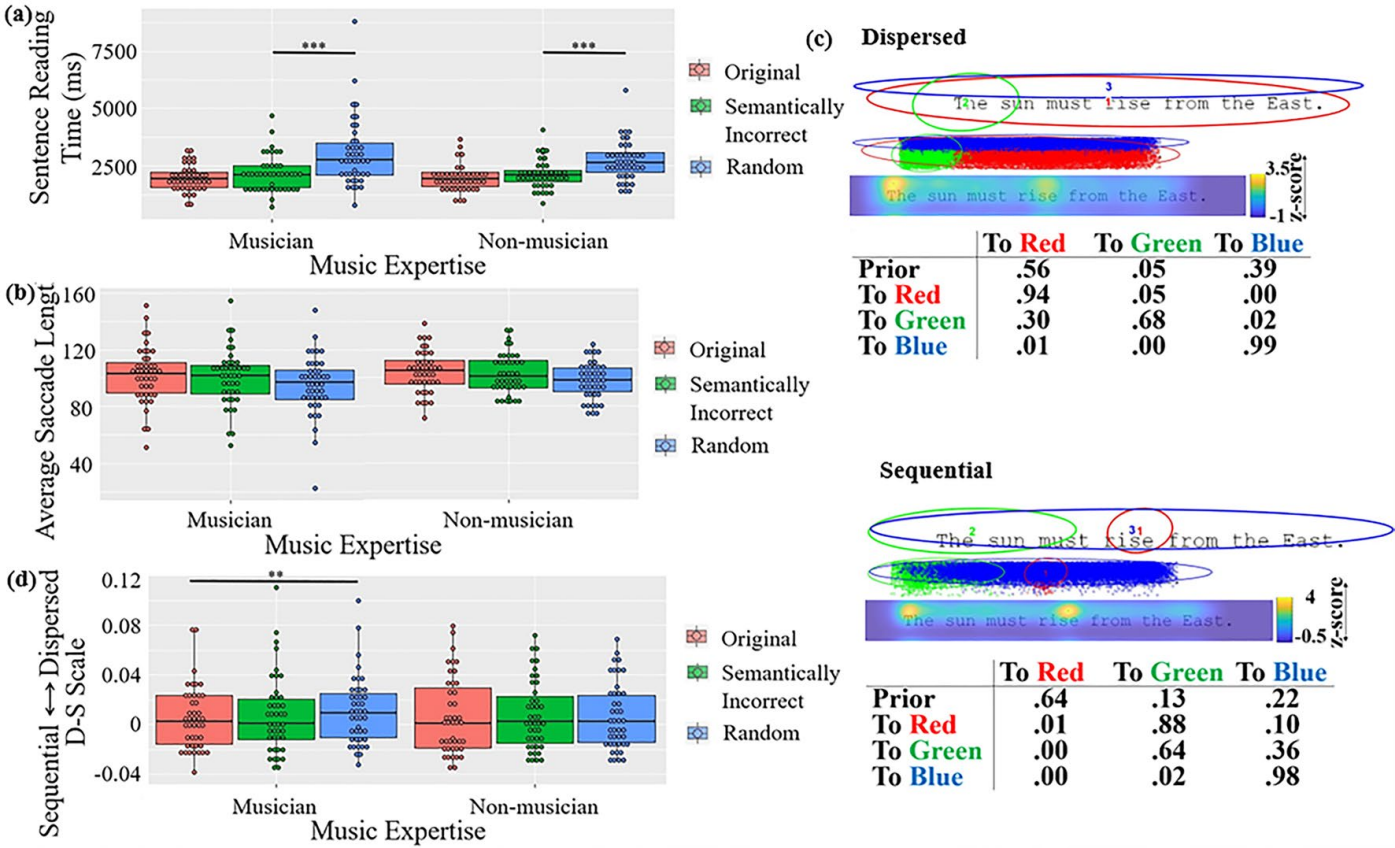
Results for English reading: (**a**) Reading time. (**b**) Average saccade length. (**c**) Two representative eye movement patterns discovered using EMHMM. Ellipses show ROIs as 2-D Gaussian emissions; the border of the ellipses show two standard deviations from the mean. The table shows transition probabilities among the ROIs. Priors show the probabilities that a fixation sequence starts from the ellipse. The smaller images show the assignment of actual fixations to different ROIs and the corresponding heatmap. The assignment of fixations to the ROIs was based on the ROI sequence with the largest posterior probability given the fixation sequence. (**d**) Eye movement pattern measured in D-S scale (***p < .001, **p < .01, *p < .05).

In reading time, an interaction between music expertise and sentence type was found, *F1*(2, 166) = 3.10, *p* = 0.048, η_p_^2^ = 0.036, *F2*(2, 69) = 20.2, *p* < 0.001, η_p_^2^ = 0.370 (Fig. 2a). Participants spent the most time reading random word lists and least time reading original sentences; this effect was stronger in musicians. No main effect of music expertise or sentence type was found. The planned comparisons for semantic processing (original vs. semantically incorrect) and linguistic regularity (original vs. random word list) showed no main effect or interaction. In syntactic processing (semantically incorrect vs. random word), an interaction between sentence type and music expertise was observed, *F1*(1, 83) = 4.06, *p* = 0.047, η_p_^2^ = 0.047, *F2*(1, 46) = 20.8, *p* < 0.001, η_p_^2^ = 0.312: the reading time difference between the two conditions was larger in musicians than non-musicians. Thus, musicians’ reading fluency was more affected by syntactic irregularities than non-musicians.

In reading efficacy, musicians and non-musicians did not differ significantly in accuracy or RT of question answering for any sentence type.

In saccade length, no significant effect was observed (Fig. 2b).

In fixation duration, a main effect of sentence type was found, *F1*(2, 166) = 4.275, *p* = 0.015, η_p_^2^ = 0.049; however the effect was not significant in by-item analysis, *F2*(2, 69) = 0.014, *p* = 0.986. In the planned comparisons, no effect was observed in semantic processing or linguistic regularity comparisons. In syntactic processing (semantically incorrect vs. random word), a main effect of sentence type was observed, *F1*(1, 83) = 7.94, *p* = 0.006, η_p_^2^ = 0.087, but the effect was not significant in by-item analysis, *F2*(1, 46) = 0.031, *p* = 0.861: the fixation duration was longer in random word lists than semantically incorrect sentences.

In regression rate, no significant effect was observed.

In skipping rate, a main effect of sentence type was observed, *F1*(2, 166) = 3.303, *p* = 0.039, η_p_^2^ = 0.038, but the effect was not significant in by-item analysis, *F2*(2, 69) = 0.249, *p* = 0.780: participants had higher skipping rate when reading original sentences than random word lists. In the planned comparisons, no effect was observed in syntactic processing or linguistic regularity comparisons. In semantic processing (original vs. semantically incorrect), a main effect of sentence type was observed, *F1*(1, 83) = 7.16, *p* = 0.009, η_p_^2^ = 0.079, but the effect was not significant in by-item analysis, *F2*(1, 46) = 20.8, *p* < 0.001, η_p_^2^ = 0.312: participants had higher skipping rate when reading original sentences than semantically incorrect sentences.

In eye movement pattern, the two representative patterns discovered by the EMHMM approach were shown in Fig. 2c. In the first pattern, a scan path typically started with a fixation at a widely distributed region covering the whole sentence (Red, 56%), and then remained in this region, with a small probability to move to the sentence beginning (Green, 5%). In contrast, in the second pattern, a scan path typically started at the middle (Red, 64%). Then, the second fixation was most likely at the sentence beginning (Green, 88%), and continued to the rest the sentence. Note that the tendency to start with a fixation at the sentence centre before moving to the sentence beginning may be related the central fixation bias reported in the literature, where participants have a tendency to make an initial fixation towards the centre of a visual stimulus regardless of its feature distribution, and this central bias may be due to its optimality for early information processing or convenience for visual exploration^52^.

Since EMHMM uses a data-driven method to discover ROIs, the widely distributed ROIs in the first pattern (Red and Blue ROIs) indicated that participants’ eye fixations did not target on specific local regions of a sentence, in contrast to the small ROIs discovered in the second pattern that were typically visited in a specific order. Accordingly, we referred to the first pattern as the dispersed pattern and the second pattern as the sequential pattern. The two patterns were significantly different, as the data log-likelihoods of the dispersed patterns given the representative dispersed HMM were significantly higher than those given the representative sequential HMM, *t*(110) = 15.947, *p* < 0.001, *d* = 1.514, and vice versa for the sequential patterns, *t*(146) = 12.146, *p* < 0.001, *d* = 1.002^34^. To quantify participants’ eye movement pattern along the dispersed-sequential pattern dimension, following previous studies^40,45,47^, we defined D-S scale as (D – S)/(|D| +|S|), where D refers to the log-likelihood of the eye movement data being generated by the representative dispersed pattern HMM, and S for the representative sequential pattern HMM. A more positive value indicated higher similarity to the dispersed pattern.

In D-S scale, ANOVA showed no significant effect. In the planned comparisons, no effect was observed in semantic or syntactic processing comparisons. In linguistic regularity (original vs. random word), an interaction between sentence type and music expertise was observed, *F1*(1, 83) = 4.430, *p* = 0.038, η_p_^2^ = 0.051, *F2*(1, 46) = 4.820, *p* = 0.033, η_p_^2^ = 0.015. Musicians showed a more sequential pattern when reading original sentences than random word lists, *t1*(83) = -3.297, *p* = 0.008, *d* = 3.317, *t2*(46) = -3.699, *p* = 0.003, *d* = 1.069, whereas non-musicians did not, *t1*(83) = -0.278, *p* = 0.992, *t2*(46) = 0.332, *p* = 0.987, *n.s*. (Fig. 2d). This suggested that musicians’ eye movement planning behaviour was affected more by linguistic (semantic and syntactic) irregularities than non-musicians.

### Chinese sentences

**Figure 3:**
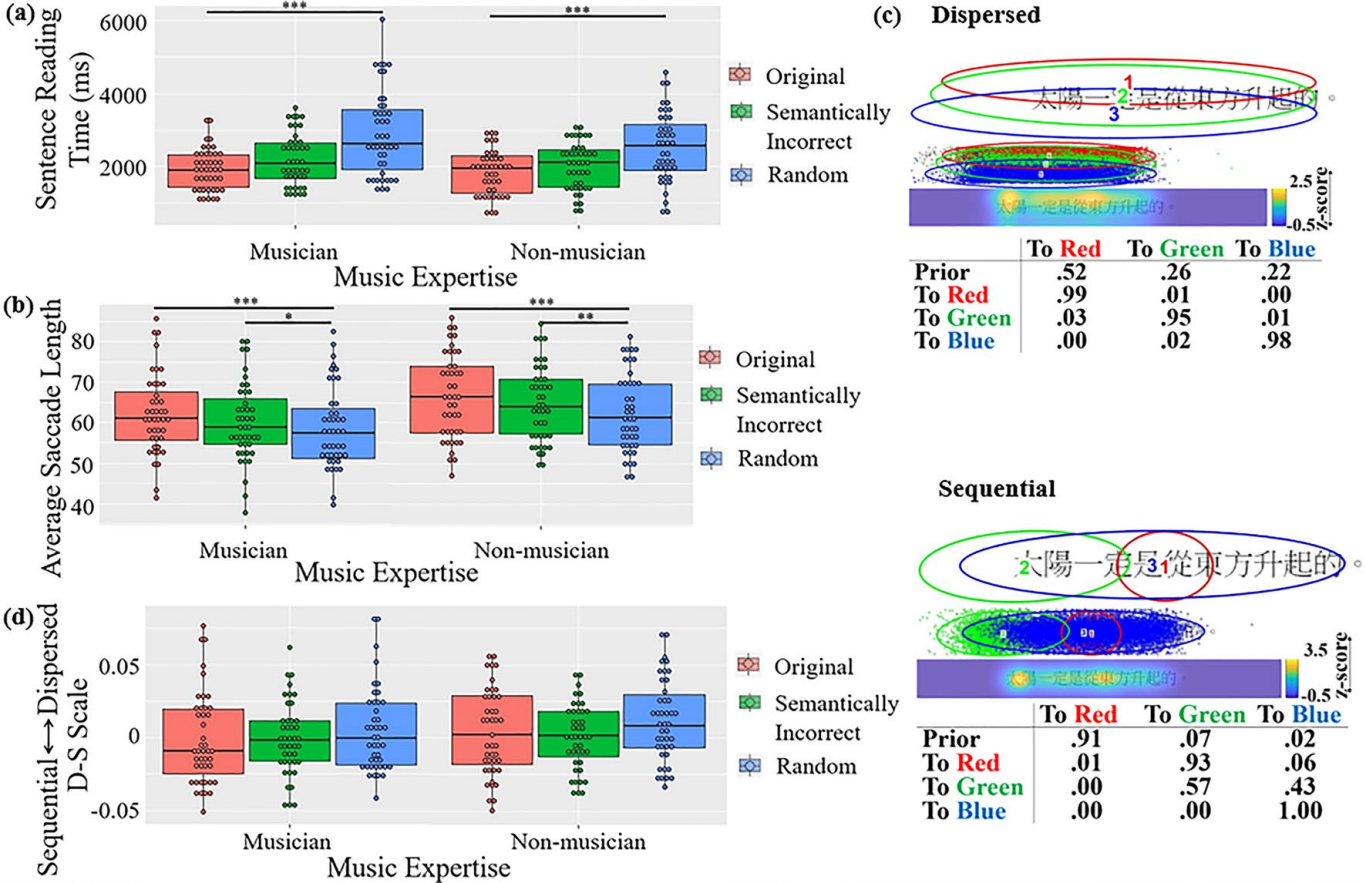
Results of Chinese reading: (**a**) Reading time. (**b**) Average saccade length. (**c**) Two common patterns discovered using EMHMM. (**d**) Eye movement pattern measured in D-S scale (***p < .001, **p < .01, *p < .05).

In reading time, similar to the English reading results, an interaction between music expertise and sentence type was found, *F1*(2, 166) = 3.41, *p* = 0.035, η_p_^2^ = 0.039, *F2*(2, 69) = 16.9, *p* < 0.001, η_p_^2^ = 0.329 (Fig. 3a): musicians spent longest time reading random word lists, and shortest time reading original sentences, whereas non-musicians spent longer time reading random word lists than semantically incorrect and original sentences. No main effect of sentence type or music expertise was observed. In the planned comparisons, no effect was observed in semantic processing (original vs. semantically incorrect) or syntactic processing (semantically incorrect vs. random word), whereas in linguistic regularity (original vs. random word) an interaction between sentence type and music expertise was observed, *F1*(1, 83) = 4.05, *p* = 0.047, η_p_^2^ = 0.047, *F2*(1, 46) = 26.5, *p* < 0.001, η_p_^2^ = 0.366: the sentence type effect was stronger in musicians than non-musicians. Thus, musicians’ Chinese reading time was more affected by linguistic regularity than non-musician.

In reading efficacy, musicians and non-musicians did not differ in accuracy or RT of question answering for any sentence type.

In average saccade length (Fig. 3b), a main effect of sentence type was observed, *F1*(2, 166) = 5.170, *p* = 0.007, η_p_^2^ = 0.059, *F2*(2, 69) = 13.2, *p* < 0.001, η_p_^2^ = 0.277: participants had longest saccade lengths when reading original sentences, and shortest when reading random word lists. In the planned comparisons, in semantic processing (original vs. semantically incorrect), no effect was observed. In linguistic processing (original vs. random word), a main effect of sentence type was observed, *F1*(1, 83) = 7.790, *p* = 0.007, η_p_^2^ = 0.086, *F2*(1, 46) = 30.9, *p* < 0.001, η_p_^2^ = 0.402. In syntactic processing (semantically incorrect vs. random), a main effect of sentence type was observed, *F1*(1, 83) = 5.937, *p* = 0.017, η_p_^2^ = 0.067, *F2*(1, 46) = 6.42, *p* = 0.015, η_p_^2^ = 0.122.

In fixation duration, no significant effect was observed.

In regression rate, a main effect of sentence type was observed, *F1*(2, 166) = 4.468, *p* = 0.013, η_p_^2^ = 0.051, but the effect was not significant in by-item analysis, *F2*(2, 69) = 1.88, *p* = 0.161: participants had higher regression rate when reading original sentences than semantically incorrect sentences, *t1*(83) = 3.145, *p* = 0.006, *d* = 1.546, *t2*(69) = 1.543, *p* = 0.278, and random word lists, *t1*(83) = 2.952, *p* = 0.011, *d* = 4.973, *t2*(69) = 1.788, *p* = 0.181. A main effect of music expertise was also observed, *F1*(1, 83) = 6.980, *p* = 0.010, η_p_^2^ = 0.078, *F2*(1,69) = 95.820, *p* < 0.001, η_p_^2^ = 0.581: musicians had lower regression rate than non-musicians. The planned comparison for semantic processing (original vs. semantically incorrect) showed a main effect of music expertise, *F1*(1, 83) = 7.146, *p* = 0.009, η_p_^2^ = 0.079, *F2*(1, 46) = 63.441, *p* < 0.001, η_p_^2^ = 0.580, and a main effect of sentence type, *F1*(1, 83) = 4.396, *p* = 0.039, η_p_^2^ = 0.050, but this effect was not significant in by-item analysis, *F2*(1, 46) = 2.68, *p* = 0.108. The planned comparison for syntactic processing (semantically incorrect vs. random word) showed a main effect of music expertise, *F1*(1, 83) = 6.98, *p* = 0.010, η_p_^2^ = 0.078, *F2*(1, 46) = 62.830, *p* < 0.001, η_p_^2^ = 0.577. The planned comparison for linguistic regularity (original vs. random word list) showed a main effect of music expertise, *F1*(1, 83) = 6.057, *p* = 0.016, η_p_^2^ = 0.068, *F2*(1, 46) = 66.12, *p* < 0.001, η_p_^2^ = 0.590, and a main effect of sentence type, *F1*(1, 83) = 6.709, *p* = 0.011, η_p_^2^ = 0.075, but this effect was not significant in by-item analysis, *F2*(1, 46) = 3.13, *p* = 0.084.

In skipping rate, a main effect of sentence type was observed, *F1*(2, 166) = 19.13, *p* < 0.001, η_p_^2^ = 0.187, *F2*(2, 69) = 4.06, *p* = 0.021, η_p_^2^ = 0.105: participants had highest skipping rate when reading original sentences and lowest skipping rate when reading random word lists. The planned comparison for semantic processing (original vs. semantically incorrect) showed a main effect of sentence type, *F1*(1, 83) = 15.04, *p* < 0.001, η_p_^2^ = 0.153, but the effect was not significant in by-item analysis, *F2*(1, 46) = 0.832, *p* = 0.336. The planned comparison for syntactic processing (semantically incorrect vs. random word) showed a main effect of sentence type, *F1*(1, 83) = 32.70, *p* < 0.001, η_p_^2^ = 0.283, but the effect was not significant in by-item analysis, *F2*(1, 46) = 3.87, *p* = 0.055. The planned comparison for linguistic regularity (original vs. random word) showed a main effect of sentence type, *F1*(1, 83) = 6.83, *p* = 0.011, η_p_^2^ = 0.076, *F2*(1, 46) = 6.76, *p* = 0.012, η_p_^2^ = 0.128.

In eye movement pattern (Fig. 3c), the disperse pattern typically started with a fixation at a widely distributed region covering the whole sentence, and then remained in this region. The sequential pattern typically started at the middle (Red, 91%), and then to the sentence beginning (Green, 93%); then continued to the rest the sentence. The two patterns were significantly different: the data log-likelihoods of the dispersed pattern given the representative dispersed HMM were significantly higher than those given the representative sequential HMM, *t*(165) = 13.1391, *p* < 0.001, *d* = 1.0198; vice versa for the data log-likelihoods of the sequential patterns, *t*(91) = 14.9432, *p* < 0.001, *d* = 1.5579.

In D-S scale, no significant effect was found. Also, no effect was found in the planned comparisons (Fig. 3d).

### Musical phrases

**Figure 4:**
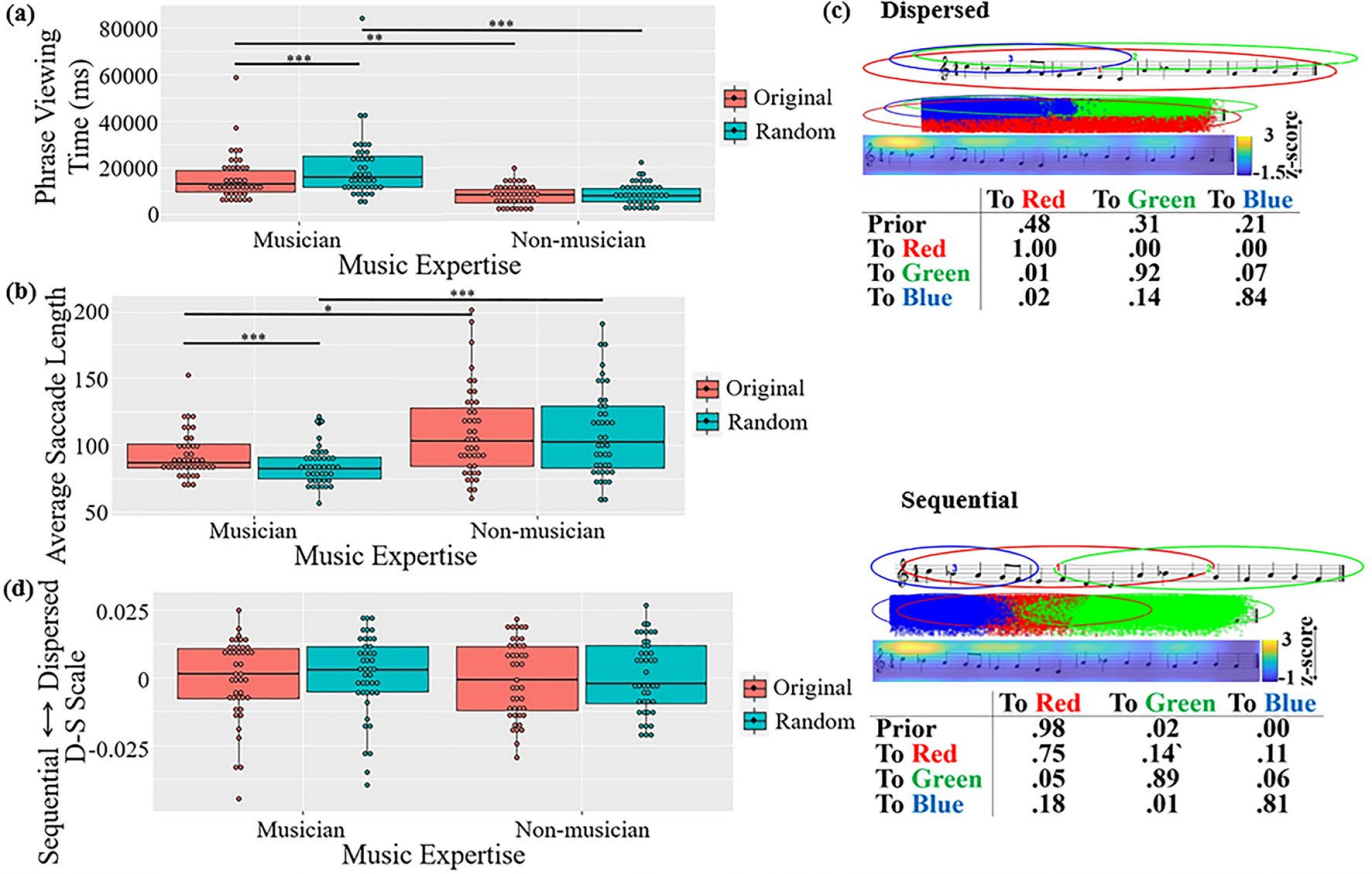
Results of the musical phrase viewing task: (**a**) Viewing time. (**b**) Average saccade length. (**c**) Two common patterns discovered using EMHMM. (**d**) Eye movement pattern measured in D-S scale (***p < .001, **p < .01, *p < .05).

In viewing time, a main effect of music expertise was observed, *F1*(1, 83) = 21.516, *p* < 0.001, η_p_^2^ = 0.206, *F2*(1, 46) = 896.1, *p* < 0.001, η_p_^2^ = 0.951; this effect interacted with sentence type, *F1*(1, 83) = 20.650, *p* < 0.001, η_p_^2^ = 0.199, *F2*(1, 46) = 35.1, *p* < 0.001, η_p_^2^ = 0.433 (Fig. 4a). Musicians spent more time viewing random segments than original phrases, *t1*(83) = 7.64, *p* < 0.001, *d* = 0.922, *t2*(46) = 6.53, *p* < 0.001, *d* = 1.886; this was not observed in non-musicians, *t1*(83) = 1.12, *p* = 0.679, *n.s*., *t2*(46) = 3.51, *p* = 0.005, *d* = 1.013.

In viewing efficacy, musicians had higher accuracy than non-musicians in the recognition of original phrases, *t1*(84) = 7.81, *p* < 0.001, *d* = 1.685, *t2*(23) = 4.20, *p* < 0.001, *d* = 0.857, and random segments, *t1*(84) = 4.94, *p* < 0.001, *d* = 1.065, *t2*(23) = 4.01, *p* < 0.001, *d* = 0.819. Musicians also had longer RT than non-musicians for original phrases, *t1*(84) = 2.48, *p* = 0.015, *d* = 0.536, *t2*(23) = 4.85, *p* < 0.001, *d* = 0.990, and random segments, *t1*(84) = 2.75, *p* = 0.007, *d* = 0.593, *t2*(23) = 7.49, *p* < 0.001, *d* = 1.528. In auditory musical phrase matching, musicians had higher accuracy than non-musicians for both original phrases, *t1*(84) = 9.282, *p* < 0.001, *d* = 2.002, *t2*(23) = 5.50, *p* < 0.001, *d* = 1.123, and random segments, *t1*(84) = 5.858, *p* < 0.001, *d* = 1.263, *t2*(23) = 4.36, *p* < 0.001, *d* = 0.891. They did not differ in RT.

In saccade length (Fig. 4b), a main effect of music expertise was observed, *F1*(1, 83) = 14.11, *p* < 0.001, η_p_^2^ = 0.145, *F2*(1, 46) = 181.53, *p* < 0.001, η_p_^2^ = 0.798. An interaction between sentence type and music expertise was also observed, *F1*(1, 83) = 10.37, *p* = 0.002, η_p_^2^ = 0.111, *F2*(1, 46) = 9.97, *p* = 0.003, η_p_^2^ = 0.178: musicians had longer saccade lengths when viewing original phrases than random segments, *t1*(83) = 5.319, *p* < 0.001, *d* = 4.337, *t2*(46) = 3.519, *p* = 0.005, *d* = 1.016, whereas non-musicians did not, *t1*(83) = 0.701, *p* = 0.896, *t2*(46) = 0.262, *p* = 0.994, *n.s*. Thus, musicians were more sensitive to irregularities in music reading reflected in average saccade length.

In fixation duration, an interaction between sentence type and music expertise was observed, *F1*(1, 83) = 14.06, *p* < 0.001, η_p_^2^ = 0.145, *F2*(1, 46) = 19.188, *p* < 0.001, η_p_^2^ = 0.294: musicians had shorter fixation duration when viewing original musical phrases than random segments, *t1*(83) = -6.005, *p* < 0.001, *d* = −5.784, *t2*(46) = −8.34, *p* < 0.001, *d* = −2.407, whereas non-musicians did not, *t1*(83) = −0.672, *p* = 0.907, *t2*(46) = −3.95, *p* = 0.357, *n.s*. Thus, musicians were more sensitive to irregularities in music reading than non-musicians as reflected in fixation duration.

In regression rate, a main effect of music expertise was observed, *F1*(1, 83) = 29.10, *p* < 0.001, η_p_^2^ = 0.260, *F2*(1, 46) = 455.38, *p* < 0.001, η_p_^2^ = 0.908: musicians had lower regression rate than non-musicians.

In skipping rate, no significant effect was observed.

In eye movement pattern (Fig. 4c), the dispersed pattern typically started with a fixation at a widely distributed region (Red and Green, 79%); then remained in these regions. The sequential pattern typically started with a fixation located at the first three bars (Red, 100%), and then stayed in the same region, with a small probability to continue to the rest of the phrase (Green, 12%), or move to the phrase beginning (Blue, 10%). The two patterns were significantly different: Data log-likelihoods of the dispersed patterns given the representative dispersed HMM were significantly higher than those given the representative sequential pattern HMM, *t*(91) = 8.14542, *p* < 0.001, *d* = 0.849, and vice versa for the data log-likelihoods of sequential patterns, *t*(79) = 11.7085, *p* < 0.001, *d* = 1.309. In D-S scale, no effect was observed (Fig. 4d).

Here we observed musicians’ sensitivity to irregularities in music reading reflected in both viewing time and saccade length. In a separate analysis, we calculated normalized viewing time difference between original and random segment conditions as (O − R)/(O + R), where O and R refer to viewing time in the original and random segment condition respectively (*r*_*SB*_ = 0.99). We found that musicians’ viewing time difference was associated with their auditory musical phrase matching accuracy: higher accuracy was correlated with longer viewing time for random segments relative to original notations, *r*(41) = −0.409, *p* = 0.006. Similarly, in musicians, larger normalized saccade length difference between original and random segment conditions (*r*_*SB*_ = 0.97) was associated with higher auditory musical phrase matching accuracy, *r*(41) = 0.335, *p* = 0.028, and smaller normalized fixation duration difference between original and random segment conditions (*r*_*SB*_ = 0.97) was associated with higher auditory musical phrase matching accuracy, *r*(41) = −0.400, *p* < 0.001. These results suggested that the viewing time, saccade length, and fixation duration effects in musicians were related to their expertise in matching music notations to corresponding auditory musical phrases.

### Tibetan sentences

**Figure 5:**
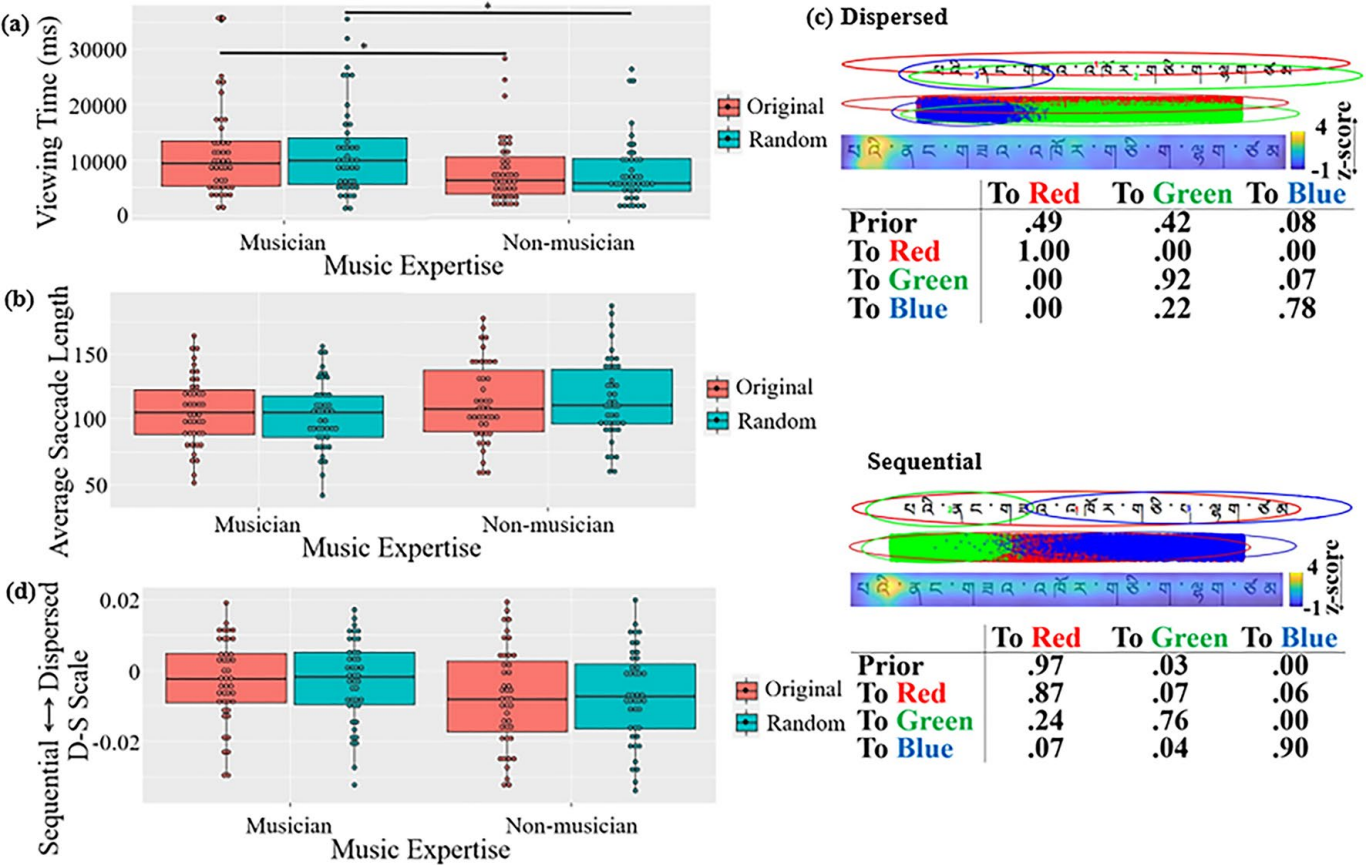
Results of Tibetan stimuli: (**a**) Viewing time. (**b**) Average saccade length. (**c**) Two common patterns discovered using EMHMM. (**d**) Eye movement pattern measured in D-S scale (***p < .001, **p < .01, *p < .05).

In viewing time, a main effect of music expertise was found, *F1*(1, 83) = 4.505, *p* = 0.037, η_p_^2^ = 0.051, *F2*(1, 46) = 646.56, *p* < 0.001, η_p_^2^ = 0.934 (Fig. 5a): musicians spent more time viewing than non-musicians. No main effect of sentence type was observed. In viewing efficacy, musicians and non-musicians did not differ in accuracy or RT of word recognition of any sentence type.

In saccade length (Fig. 5b), an interaction between sentence type and expertise was observed, *F1*(1, 83) = 12.419, *p* < 0.001, η_p_^2^ = 0.130, *F2*(1, 46) = 9.90, *p* = 0.003, η_p_^2^ = 0.177: musicians had marginally longer average saccade lengths when viewing original sentences than random syllable lists, *t1*(83) = 2.513, *p* = 0.065, but the effect was not significant in by-item analysis, *t2*(46) = 2.25, *p* = 0.125; whereas non-musicians had marginally shorter average saccade lengths when viewing original sentences than random word lists, *t1*(83) = −2.541, *p* = 0.061, and the effect was not significant in by-item analysis, *t2*(46) = −2.10, *p* = 0.168.

No significant effect was observed in fixation duration, regression rate, or skipping rate.

In eye movement pattern (Fig. 5c), the disperse pattern typically started with a fixation at a widely distributed region (Red and Green, 92%); then remained in these regions. The sequential pattern typically started with a fixation at a widely distributed region (Red, 98%), and then had a small probability to move to the end (Blue, 7%) or the sentence beginning (Green, 8%). The two patterns were significantly different (Data log-likelihoods of the dispersed patterns given the representative dispersed HMM were significantly higher than those given the representative sequential HMM, *t*(72) = 15.7654, *p* < 0.001, *d* = 1.8452; vice versa for the sequential patterns, *t*(98) = 4.75833, *p* < 0.001, *d* = 0.4782). In D-S scale, no significant effect was observed (Fig. 5d).

## Discussion

Here we tested the hypothesis that in Chinese-English bilinguals, music reading experience may modulate eye movement planning in reading English and Chinese sentences differentially due to higher similarity in the perceptual processes involved between music and English reading than between music and Chinese reading. Consistent with our hypothesis, we found that Chinese-English bilingual musicians’ overall eye movement pattern (as measured along the dispersed-sequential dimension using EMHMM) in reading English sentences was disturbed by syntactic violations whereas non-musicians’ eye movement planning behaviour was not. A similar phenomenon was observed in music notation reading, although the effect was in saccade length instead of overall eye movement pattern. In contrast, this sensitivity to syntactic violations was not observed in either overall eye movement pattern or saccade length during Chinese sentence reading, and musicians and non-musicians did not differ in these eye movement planning measures in Chinese sentence reading.

Consistent with these findings, previous studies found that Chinese-English bilingual musicians had a larger visual span for English letter identification and better English word naming performance than non-musicians, but these effects were not observed in their Chinese character identification or naming. These phenomena were argued to be because both English and music notation reading involve mapping individual visual components to individual sounds from left to right (i.e., grapheme-phoneme mapping in reading English words and note-to-pitch mapping in reading musical segments) with spacing between words/music segments^5,6^. Here we further showed that as compared with non-musicians, Chinese-English bilingual musicians’ eye movement planning behaviour was affected by syntactic violations in reading both English sentences and music notations, but not in reading Chinese sentences. This result suggests that the similarity in perceptual processes involved between English and music notation reading may also modulate eye movement planning for syntactic processing. Note that in the current study, musicians’ change in eye movement planning behaviour due to syntactic violation during English sentence reading was observed in overall eye movement pattern summarized in an HMM using the EMHMM approach, but not in average saccade length (or in any other eye movement measure in the exploratory examinations including fixation duration, regression rate, or skipping rate). This result suggested that the modulation effect of musicians’ music notation reading experience was on how participants planned where to look and the order of where to look during reading, rather than on eye movement behaviour such as saccade length, fixation duration, regression rate, or skipping rate.

Note that in contrast to eye movement planning behaviour, Chinese-English bilingual musicians’ sentence reading time was affected by syntactic violations more than non-musicians in both English and Chinese sentence reading. This result suggests that they had higher sensitivity to syntactic regularities in both English and Chinese than non-musicians. In English reading, musicians’ higher sensitivity to regularities in sentence structure reflected in reading time involved mainly syntactic regularities, as it was found in the planned comparison between semantically incorrect and random word lists. In contrast, in Chinese reading, musicians’ higher sensitivity to regularities reflected in reading time involved a combination of syntactic and semantic regularities. Previous research has suggested that musicians differed from non-musicians in linguistic processing at both semantic and syntactic levels^21,22,24^. Due to the logographic nature of Chinese orthography, Chinese sentence processing has been considered more semantics-based whereas English more syntax-based^53^. Indeed, during Chinese reading, semantic information processing from parafoveal vision was shown to precede phonological processing, suggesting that Chinese characters are optimized for semantic processing^54,55^. Also, semantic preview benefit from parafoveal vision has been more consistently reported in Chinese reading^56–58^ as compared with reading in alphabetic languages^59–63^, suggesting more semantic processing involvement during Chinese reading. Also, in Chinese, the same word may serve several different syntactic functions (For instance, the word “多” could be used as an adverbial modifier, a predicate, a verbal object, etc.^64^), and there is greater variability in word order than in English^53^. These may lead to greater reliance on semantic processing during Chinese reading than English reading. Our reading time results were consistent with these findings, showing that musicians had higher sensitivity to syntactic irregularities in English reading and higher sensitivity to a combination of syntactic and semantic irregularities in Chinese reading than non-musicians.

To better understand the mechanism underlying musicians’ and non-musicians’ difference in sensitivity to linguistic regularity, in an exploratory examination, we examined which music and language expertise factors best predicted this sensitivity. We calculated normalized sensitivity to linguistic regularity as (O—R)/(O + R), where O and R referred to the measure (either eye movement or reading time measure) in the original sentence and random word list condition respectively. Stepwise multiple regression predicting normalized sensitivity to linguistic regularity in eye movement pattern during English reading using the 5 MSIs, accuracy of the musical phrase auditory matching task and LexTALE as predictors showed that the MSI on singing abilities was the best predictor, *β* = 0.027, *p* = 0.011, with ${R}^{2}$ = 0.074, *F*(1, 84) = 6.738, *p* = 0.011. In contrast, in predicting normalized sensitivity to linguistic regularity in reading time during English reading, the MSI on emotions was the best predictor, *β* = 0.004, *p* = 0.019, with ${R}^{2}$ = 0.064, *F*(1, 84) = 5.754, *p* = 0.019. Similarly, in Chinese reading, stepwise multiple regression predicting normalized sensitivity to linguistic regularity in reading time with the 5 MSIs, accuracy of the musical phrase auditory matching task and the 7-point scale of scores in matriculation public examination of Chinese Language in Hong Kong as predictors showed that it was also best predicted by the MSI on emotions, *β* = 0.005, *p* = 0.001, with ${R}^{2}$ = 0.122, *F*(1, 84) = 11.710, *p* = 0.001. This finding suggested that the effects in reading time and eye movement pattern involved different aspects of music expertise/cognitive processes. The MSI on singing abilities measures accuracy of recalling a familiar or newly-learned song. Higher MSI on singing abilities has been reported to be associated with better performance in antisaccade, stop signal, and Stroop tests^65^, suggesting its relevance to inhibition ability and executive attention. Thus, as compared with non-musicians, musicians may engage executive attention more when resolving linguistic irregularities, resulting in larger eye movement pattern difference between the original sentence and random word list conditions. Indeed, recent research has reported a positive correlation between musical practice time and executive function abilities in adults^66^, and eye movement behaviour is related to one’s executive function and visual attention abilities^40,48^. In contrast, the MSI on emotions measures the ability to evaluate emotions that music expresses and is shown to be related to working memory abilities^65^. Thus, musicians’ increase in reading time due to linguistic violations may be related to more engagement in working memory for analysing expressions of linguistically irregular sentences. Indeed, short-term music training has been shown to improve participants’ accuracy in judging expressed emotions in speech stimuli^67^, and musicians were shown to have better sequential visual working memory than non-musicians^68^. Thus, music training may enhance engagement in sequential working memory for expression analysis, make musicians more affected by expression anomalies in language processing as presented in the random word list condition.

In musical phrase viewing, musicians were sensitive to diatonic rules as reflected in longer viewing time when viewing non-diatonic, chromatic random segment lists than diatonic musical phrases, whereas non-musicians were not. This effect was also observed in saccade length (and fixation duration), but not in overall eye movement pattern measured in EMHMM. We speculated that the stimuli used in the current study might be too short (4-bar phrases) to allow musicians to change eye movement behaviour according to diatonic rules, as violation of diatonic rules depends on the relationship across multiple musical segments/bars. Also, the task used, passive viewing with a follow-up musical segment recognition task, differed from musicians’ usual sight-reading experiences with music notations, which may have obscured their expertise. Indeed, eye movements in visual tasks are shown to be task-specific^46,69^. Similarly, previous study showed that music expertise modulated visual span for the identification of English letters but not music notes, and they speculated that this phenomenon may be because the stimuli used, random notes, differed from musicians’ usual reading experience^6^. Future work will examine these possibilities. Note that here both the effects in viewing time, saccade length, and fixation duration in musicians were associated with their accuracy in the auditory musical phrase matching task. These correlation effects were not observed when they were reading English or Chinese sentences. This result suggested that musicians’ sensitivity to music regularity during music reading was more relevant to the fluency in notation-sound mapping, and may be fundamentally different from the sensitivity effects to regularities in sentence structure observed in English or Chinese reading.

Although participants had no experience with Tibetan, musicians had longer viewing time than non-musicians, suggesting that musicians may be more motivated to analyse sentences in a novel language not learned before. Interestingly, musicians had longer saccade lengths when viewing original sentences than random word lists as compared with non-musicians. We speculated that formal music training may be associated with enhanced sensitivity to regularities in the visual stimuli when viewing sentences in a novel alphabetic language such as Tibetan. However, this effect was limited to local saccadic behaviour rather than overall pattern as measured in EMHMM, suggesting that this effect may involve different cognitive mechanisms from that observed in English sentence reading. Previous research has shown that music training may facilitate word learning in a novel language^24^. Our data further suggested that it may also modulate sentence processing in a novel language.

Together these results suggest that although musicians’ eye movement planning behaviour was more affected by syntactic violations than non-musicians’ in English sentence reading, music notation reading, and Tibetan sentence viewing, the cognitive mechanisms underlying these effects may differ. Here we found that the effect in music notation reading was associated with musicians’ expertise in notation-pitch mapping. Since the effect in English sentence reading was in overall eye movement pattern including where participants looked and the order of where they looked, it may be more related to executive function or planning ability for sentence understanding. This speculation is consistent with the exploratory analysis using MSIs reported above. In contrast, since participants had no prior knowledge of Tibetan syntactic structures, musicians’ effect in Tibetan sentence viewing may be related to the ability in analysing visual regularities in sequential structures. Indeed, music training is shown to lead to multifaceted benefits to one’s cognitive ability development, including executive function, planning, and selective attention abilities^70,71^. Thus, music expertise may be better understood as a multidimensional factor^28^, which may help us better understand the cognitive mechanisms underlying different modulation effects. Future work will examine these possibilities.

Note that in the current examination, we directly compared musicians and non-musicians to examine potential modulation effects of music reading experience on eye movement planning behaviour in bilinguals. Although we have attempted to match musicians' and non-musicians’ backgrounds as closely as possible including gender, age, and cognitive abilities, it remained possible that the differences observed between musicians and non-musicians were due to factors other than music reading experience. For example, here we found that the two groups differed in verbal working memory ability as measured in the verbal two-back task. Previous research has suggested that working memory ability is associated with one’s eye movement behaviour. For example, adult readers with worse working memory ability were found to have more regressions and longer fixation durations when reading sentences with complex structures such as those with object relative clauses^72^. Thus, to control for this difference, participants’ verbal working memory ability was added as a covariate in all our analyses reported here. The two groups may also differ in characteristics relevant to their decision to receive music training or not. In addition, although in Hong Kong children in general receive formal bilingual education starting from age 3 or younger, they may acquire the Chinese language earlier and also be exposed to Chinese more often during daily life. Thus, the differential modulation effect between English and Chinese reading may be related to participants’ experience with the two languages. Indeed, a previous study reported that while music training was correlated with Chinese children’s academic development of both L1 and L2, it did not contribute to L1 development independently from pre-training L1 performance. In contrast, music training independently contributed to L2 performance in addition to IQ and mother’s education, suggesting that musical training may influence L2 development more than L1^73^. To rule out these possibilities, future work may manipulate participants’ music reading experience through a longitudinal training study, and examine whether a similar differential modulation effect can be observed in English-Chinese bilingual musicians.

In conclusion, here we show that music reading experience may modulate eye movement planning behaviour in reading sentences in bilinguals’ two languages differentially, depending on their similarities in perceptual processing demands to music reading. Specifically, we show that Chinese-English bilingual musicians’ eye movement planning behaviour was affected by syntactic violations in both music notation and English sentence reading whereas non-musicians’ was not. In contrast, this sensitivity to syntactic violations in eye movement planning was not observed in Chinese sentence reading in either musicians or non-musicians. This difference between the two languages may be due to higher similarity between music and English reading than between music and Chinese reading in perceptual demands on processing sequential symbol strings from left to right separated by spaces. Musicians’ sensitivity to syntactic violations revealed in eye movement planning behaviour was also observed in viewing sentences in an unfamiliar alphabetic language (Tibetan). Thus, how skills in a perceptual expertise domain, such as music notation reading, can influence processes involved in other domains, such as learning to read in English, Chinese, or a novel language, depends on the similarities of the processes involved.

## Data Availability

The data that support the findings of this study are openly available in osf at https://osf.io/3xvf4/?view_only=b23b65567e2b46d6b0e7156d18262aeb.
